# Surgical Removal of Submacular Perfluorocarbon Liquid Using a 41-Gauge Extendible Subretinal Injection Needle

**DOI:** 10.18502/jovr.v14i3.4798

**Published:** 2019-07-18

**Authors:** Khalil Ghasemi Falavarjani, Pasha Anvari

**Affiliations:** ^1^Eye Research Center, Rassoul Akram Hospital, Iran University of Medical Sciences, Tehran, Iran; ^2^Department of Ophthalmology, Rassoul Akram Hospital, Iran University of Medical Sciences, Tehran, Iran

**Keywords:** Subretinal Injection Needle, Subretinal Perfluorocarbon Liquid, Vitrectomy

## Abstract

Submacular perfluorocarbon liquid (PFCL) retention is a well-known complication of vitreoretinal surgeries; however, the optimal surgical technique for the removal of subfoveal PFCL is yet to be determined. We describe a novel surgical technique for the removal of retained submacular PFCL by performing a retinotomy adjacent to the inferotemporal arcade using a 41-gauge extendible subretinal injection needle and inducing a therapeutic retinal detachment. Through the same retinotomy, the bent 41-gauge needle was advanced into the subretinal space to reach the PFCL bubble. Subsequently, active aspiration of PFCL was performed. The surgical procedure was successfully performed in two patients. This technique appears to be an effective surgical approach for removing retained submacular PFCL bubble.

##  INTRODUCTION

Submacular perfluorocarbon liquid (PFCL) retention is a rare, but well-recognized, complication of vitreoretinal surgery.^[1,2]^ It is widely accepted that subfoveal PFCL should be removed as early as possible because of its potential toxicity to photoreceptors and retinal pigment epithelium (RPE).^[3,4]^ Several surgical techniques have been proposed to manage the subfoveal PFCL.^[5,6,7]^ These techniques can be divided into two categories: displacement of PFCL by inducing therapeutic retinal detachment (RD) and postoperative positioning,^[5]^ and removal of PFCL by direct aspiration from the top or edge of the bubble.^[6,8,9,10]^ However, the visual and anatomic outcomes are variable, and the optimal surgical technique is yet to be determined.

Here, we report a novel technique to remove submacular PFCL while minimizing surgical trauma to the foveal structures.

##  SURGICAL METHOD

A standard three-port, 23-gauge vitrectomy was performed. An area without major retinal vessels adjacent to the inferotemporal vascular arcade was considered as the retinotomy site. For an easier access to the retinotomy site, the location of pars plana inflow was changed from conventional inferotemporal port to superotemporal sclerotomy.

**Figure 1:**
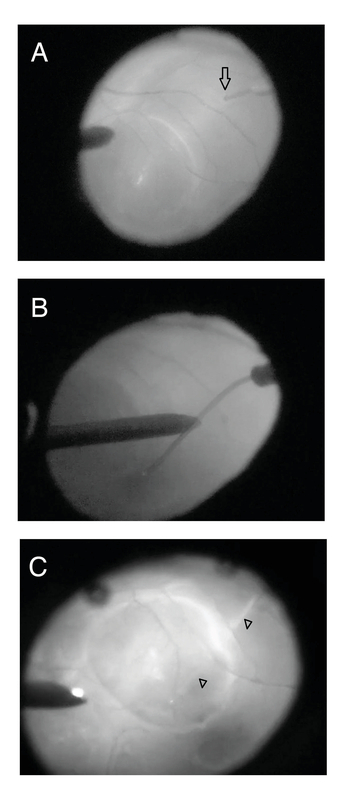
Essential surgical steps for the removal of submacular perfluorocarbon liquid (PFCL) using a 41-gauge extendible subretinal injection needle. (A) Artificial retinal detachment induction by slow injection of balanced salt solution (BSS) through the retinotomy incision near the inferotemporal arcade (arrow). (B) Bending the appropriate length of flexible needle with the aid of endoilluminator. (C) Active aspiration of PFCL bubble following needle entry into the subretinal space (arrowhead).

**Figure 2:**
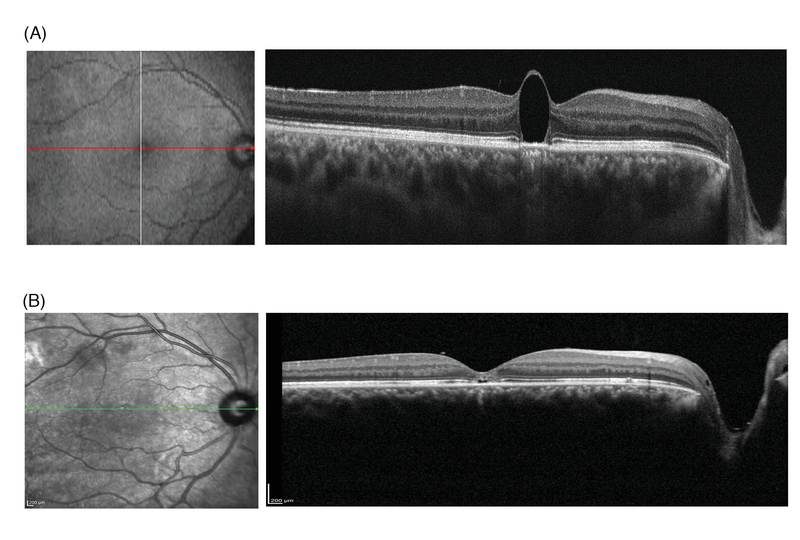
(A) Preoperative optical coherence tomography (OCT) image showing subfoveal perfluorocarbon liquid (PFCL) bubble. (B) Postoperative OCT image, three weeks after surgery showing the complete removal of PFCL bubble. Foveal ellipsoid zone and external limiting membrane disruption are also present.

**Figure 3:**
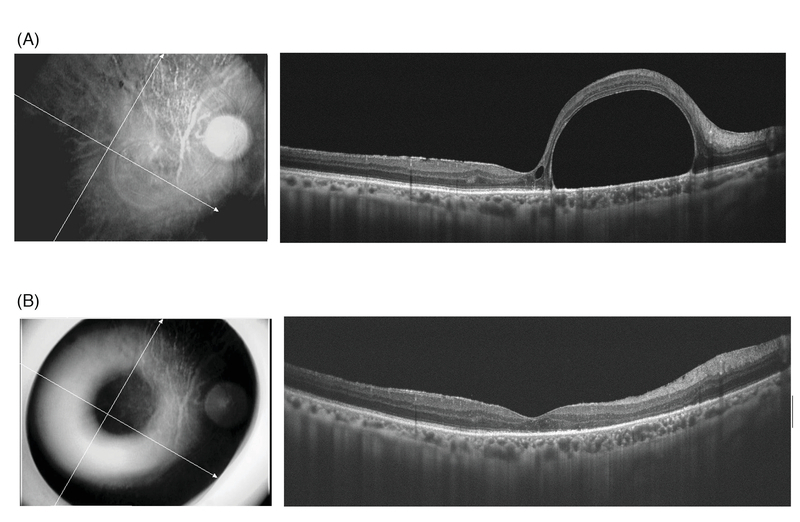
(A) Preoperative optical coherence tomography (OCT) image demonstrating large subfoveal perfluorocarbon liquid bubble, distorting normal foveal contour. (B) Postoperative OCT image, one month after surgery, confirms the complete removal of perfluorocarbon liquid bubble.

A 41-gauge extendible subretinal injection needle (D.O.R.C International, Zuidland, Netherlands) was attached to the viscous fluid injection system (Stellaris PC, Bausch + Lomb) containing a balanced salt solution (BSS). Retinotomy was performed using the tip of the 41-gauge needle. An artificial RD was induced by slow injection of BSS into the subretinal space via the retinotomy [Figure 1(A)]. The RD size was increased to completely include the subretinal PFCL bubble. The extendible needle was bent by 75${}^{\text{o}}$ with the aid of the endoilluminator probe to have adequate length to reach the PFCL bubble from the retinotomy site [Figure 1(B)]. Active aspiration pressure was tested before inserting the needle to avoid a sudden collapse of the subretinal space. The bent needle was then inserted into the retinotomy and advanced carefully in the subretinal space to reach the PFCL bubble. Afterward, active controlled aspiration (50–100 mmHg) of subfoveal PFCL was performed [Figure 1(C)]. To confirm the removal of PFCL, the aspirate was injected intravitreally, and the presence of PFCL was verified. Subsequently, retinal reattachment with PFCL or fluid–air exchange and endolaser photocoagulation of the edge of the retinotomy was performed, and 20% sulfur hexafluoride (SF6) was used as endotamponade. Video 1 demonstrates the surgical steps (supplemental digital content).

##  RESULTS

###  Case Report 1

A 19-year-old man was referred with retained subfoveal PFCL, six years after pars plana vitrectomy for macula-off rhegmatogenous retinal detachment (RRD). Best corrected visual acuity (BCVA) was counting fingers at 1 meter. The presence of PFCL was confirmed in optical coherence tomography (OCT) [Figure 2(A)]. Surgical removal of subfoveal PFCL was performed successfully using the aforementioned technique. Two months post-surgery, his BCVA improved to counting fingers at 3 meters, and OCT confirmed the complete removal of PFCL bubble [Figure 2(B)].

###  Case Report 2

A 53-year-old man was referred with retained subfoveal PFCL, three months following silicone oil removal. Silicone oil tamponade had been performed during pars plana vitrectomy for RRD. BCVA was 20/200, and OCT imaging confirmed the presence of PFCL [Figure 3(A)]. Submacular PFCL was removed using the same technique as case 1. Complete removal of PFCL was verified using OCT [Figure 3(B)]. Two months post-operation, his BCVA improved to 20/70.

##  DISCUSSION

Although some studies have reported a stable course of small retained subretinal PFCL,^[12,13]^ many authors advocate its early removal,^[14,15]^ as growing evidence suggest irreversible damage of photoreceptors and RPE cells due to direct PFCL toxicity or altered retinal metabolism.^[4,16,17]^ In addition, our experience with the first patient demonstrated that even in long-standing cases, there is still a chance of improvement in visual acuity following surgical removal of PFCL.

Several studies have reported the results of subfoveal PFCL removal.^[5,6,7][18]^ The retinotomies have generally been performed in the foveal area, either on the top of the PFCL bubble or adjacent to it. Consequently, the anatomical and visual prognoses were guarded. Our technique has the advantage of approaching subfoveal PFCL from a site distant to the fovea, minimizing the risks associated with direct aspiration over or adjacent to the subfoveal PFCL bubble, including damage to the foveal photoreceptors and RPE cells, and subfoveal hemorrhage. We selected the extramacular location to avoid accumulation of blood in the subfoveal area in case of accidental hemorrhage at the retinotomy site. In the first case, we created the retinotomy in the superotemporal location [Figure 2], however, for the second patient, the inferotemporal location was selected [Figures 1 and 3]. If small bubbles of PFCL remain, the inferior location of the RD will facilitate inferior displacement of the bubble with postoperative positioning.^[5]^ Laser photocoagulation of the retinotomy edge is easy and safe in an extramacular location.

The induced RD should be high enough to allow an easy movement of the bent needle in the subretinal space, avoiding any trauma to the overlying retina and underlying RPE. Nevertheless, it is essential to gently inject BSS, as the thinned retinal tissue overlying PFCL bubble is susceptible to rupture, and high injection pressure may lead to macular hole formation.^[19]^

In conclusion, our novel technique appears to be an effective surgical approach for removing retained subfoveal PFCL. Larger studies are needed to confirm the safety of this approach.

##  Financial Support and Sponsorship

Nil.

##  Conflicts of Interest

There are no conflicts of interest.
